# The Impact of Different Oral Antidiabetic Drugs on Insulin Pump Intensive Therapy in Type 2 Diabetes Patients: A Clinical Study

**DOI:** 10.1155/jdr/9957473

**Published:** 2026-03-31

**Authors:** Miaoguan Peng, Yijuan Xie, Naifeng Liang, Shiyun Wen, Yaojie Zhai, Yingjun Xie, Yuyi Chen

**Affiliations:** ^1^ Department of Endocrinology, The Third Affiliated Hospital, Guangzhou Medical University, Guangzhou, 510150, China, gzhmc.edu.cn; ^2^ The Affiliated Brain Hospital, Guangzhou Medical University, Guangzhou, 510170, China, gzhmc.edu.cn; ^3^ Guangdong Provincial Key Laboratory of Major Obstetric Diseases, Guangzhou Medical University, Guangzhou, 510150, China, gzhmc.edu.cn; ^4^ Guangdong Provincial Clinical Research Center for Obstetrics and Gynecology, Guangzhou Medical University, Guangzhou, 510150, China, gzhmc.edu.cn; ^5^ Guangdong-Hong Kong-Macao Greater Bay Area Higher Education Joint Laboratory of Maternal-Fetal Medicine, Guangzhou Medical University, Guangzhou, 510150, China, gzhmc.edu.cn; ^6^ Key Laboratory of Neurogenetics and Channelopathies of Guangdong Province and the Ministry of Education of China, Guangzhou Medical University, Guangzhou, 510170, China, gzhmc.edu.cn; ^7^ The Third Clinical College of Guangzhou Medical University, The Third Affiliated Hospital, Guangzhou Medical University, Guangzhou, 510150, China, gzhmc.edu.cn; ^8^ The Third Affiliated Hospital, Guangzhou Medical University, Guangzhou, 510150, China, gzhmc.edu.cn; ^9^ Department of Obstetrics and Gynecology, The Third Affiliated Hospital of Guangzhou Medical University, No. 63 of Duobao Road, Guangzhou, 510150, Guangdong, China, gzhmc.edu.cn; ^10^ Department of Obstetrics, The Third Affiliated Hospital of Guangzhou Medical University, No. 63 of Duobao Road, Guangzhou, 510150, Guangdong, China, gzhmc.edu.cn

**Keywords:** dapagliflozin, glycaemic control, insulin pump therapy, metformin, pancreatic function, T2DM

## Abstract

**Objective:**

This study aimed to compare the effects of insulin pump intensive therapy alone with those of insulin pump intensive therapy combined with metformin or dapagliflozin on the time to achieve glycemic targets, total insulin dose at the end of intensive therapy, and pancreatic function. The study aimed to understand the impact of adding the traditional antidiabetic drug metformin and the novel antidiabetic drug dapagliflozin to routine intensive insulin therapy on treatment outcomes.

**Methods:**

A total of 110 patients with newly diagnosed T2DM (with glycated hemoglobin >9%, fasting blood glucose >11.1 mmol/L, or obvious symptoms of hyperglycemia) were enrolled in a single‐center randomized controlled trial. Patients were randomized into three groups. The final analysis included 81 participants (73.6% of the enrolled) with complete data for the primary outcomes, distributed as follows: Group 1 (insulin only, *n* = 25), Group 2 (insulin plus dapagliflozin, *n* = 20), and Group 3 (insulin plus metformin, *n* = 36). The term “newly diagnosed” was defined as a diagnosis confirmed within 1 year prior to enrollment, in accordance with the Chinese guidelines for the prevention and treatment of T2DM. All patients were either treatment‐naïve or had not received any sustained glucose‐lowering pharmacotherapy for more than 2 weeks since diagnosis. The primary outcomes were the time to achieve glycemic targets, total insulin dose at the end of intensive therapy, and pancreatic function.

**Results:**

The fasting insulin levels in all the treatment groups changed over time, with greater decreases observed in the insulin pump therapy alone group and the insulin pump therapy + dapagliflozin group than in the insulin pump therapy + metformin group.

**Conclusion:**

These findings suggest that compared with metformin, dapagliflozin combined with intensive insulin pump therapy holds greater promise for optimizing glycaemic control in patients with T2DM, thus providing a more promising option for clinical practice.

## 1. Introduction

Diabetes mellitus is a chronic metabolic disorder characterized by persistently elevated blood glucose levels, which are predominantly attributed to either insufficient insulin secretion or insulin resistance [1]. Between 2007 and 2017, the prevalence of diabetes in China, as diagnosed according to the World Health Organization (WHO) criteria, significantly increased, with the prevalence increasing from 39.4% to 53.6% among adults over 10 years of age (*p* = 0.0004) [2]. The diagnostic criteria for type 2 diabetes (T2DM) encompass the manifestation of characteristic diabetic symptoms—namely polydipsia, polyphagia, polyuria, and unexplained weight loss—combined with biochemical evidence of hyperglycemic. Specifically, diagnosis is established when random blood glucose concentrations are equal to or exceed 11.1 mmol/L, fasting blood glucose levels are equal to or greater than 7.0 mmol/L, or 2‐h postload glucose measurements reach or surpass 11.1 mmol/L [3]. Several studies have demonstrated that for individuals who are newly diagnosed with diabetes or those with long‐standing diabetes and suboptimal glycaemic control, short‐term intensive insulin therapy can mitigate the damage to pancreatic islet cells caused by hyperglycemic toxicity and can lead to clinical remission for more than 1 year in nearly half of patients [2].

Intensive insulin therapy is a cornerstone of glycemic control, and it involves the administration of insulin multiple times daily (typically 3–4 times) via subcutaneous injection or continuous subcutaneous insulin infusion (CSII) using an insulin pump in conjunction with dietary and exercise therapy [3]. China’s clinical expert guidelines for short‐term intensive insulin therapy in T2DM recommend this approach for two specific scenarios: (1) newly diagnosed T2DM patients with marked hyperglycemia (defined as glycated hemoglobin [HbA1c] >9%, fasting plasma glucose [FPG] >11.1 mmol/L, or overt hyperglycemic symptoms); and (2) T2DM patients with a certain disease duration who are on combination therapy with two or more oral antidiabetic drugs (OADs) but still have significantly elevated HbA1c (>9%) and need continued T2DM treatment [4]. In patients who have been treated with two or more oral hypoglycemic agents but continue to exhibit significant hyperglycemia (HbA1c >9%) or fail to achieve target glucose levels despite adequate dose adjustments (HbA1c >7.0%), short‐term intensive insulin therapy may be considered. During this therapy, insulinotropic agents are discontinued, while oral antidiabetic agents, such as metformin or α‐glucosidase inhibitors, can be continued [5, 6].

Metformin, which is a long‐standing oral hypoglycemic agent, remains a first‐line treatment for T2DM, as evidenced by recent guidelines from China and the AACE/ACE [7]. Metformin is well documented for its efficacy, safety, cost‐effectiveness, and cardiovascular benefits. Expert consensus supports its use to enhance insulin sensitivity and maintain glycemic control—particularly during the transition from oral antidiabetic agents to insulin [8]. The HOME study—a 4.3‐year prospective, randomized, double‐blind, and placebo‐controlled trial—demonstrated that metformin combined with insulin enhances glucose‐lowering efficacy, reduces daily insulin dosage (by an average of 19.63 U), and mitigates weight gain in patients with T2DM [9]. Dapagliflozin, which member of the SGLT2 inhibitor class, exerts its primary effect by targeting the luminal membrane of the proximal renal tubule, thereby inhibiting glucose reabsorption via SGLT2 blockade, lowering renal glucose reabsorption thresholds, and increasing urinary glucose excretion to reduce blood glucose levels [10, 11]. This hypoglycemic mechanism does not depend on insulin secretion or action, and therefore exerts a certain hypoglycemic effect in patients with T2DM, regardless of their pancreatic function.

The 2017 AACE/ACE Consensus Statement on the Comprehensive Management of T2DM advocated lifestyle modifications combined with a single antidiabetic agent, such as metformin, as the initial treatment for patients with newly diagnosed T2DM or mild hyperglycemia (HbA1c <7.0%) [12]. For the management of T2DM, a range of OADs are available, including glucagon‐like peptide‐1 (GLP‐1) receptor agonists, dipeptidyl peptidase‐4 (DPP‐4) inhibitors, sodium‐glucose cotransporter‐2 (SGLT2) inhibitors, thiazolidinediones (TZDs), and alpha‐glucosidase inhibitors combined with sulfonylureas. The American Diabetes Association (ADA) recommends sulfonylureas, TZDs, DPP‐4 inhibitors, SGLT2 inhibitors, and GLP‐1 receptor agonists; in contrast, the 2017 Consensus Statement by the American Association of Clinical Endocrinologists/American College of Endocrinology (AACE/ACE) emphasizes prioritizing medications with significant weight‐loss effects (e.g., SGLT2 inhibitors, GLP‐1 receptor agonists) while advocating cautious use of insulin and its analogy. For patients with glycated hemoglobin (HbA1c) <9.0% who present with diabetic symptoms (e.g., polydipsia, polyuria, polyphagia, and weight loss), insulin therapy is often beneficial—especially when combined with long‐acting insulin or intensive treatment strategies (e.g., add‐on of GLP‐1 receptor agonists, SGLT2 inhibitors, DPP‐4 inhibitors, or mealtime insulin) [12].

The 2018 AACE/ACE Comprehensive Management Strategy for T2DM reinforces the central role of metformin while highlighting the preference for newer drugs with weight reduction and cardiovascular benefits, such as SGLT2 inhibitors and GLP‐1 receptor agonists [7]. In a review published in the Expert Review of Clinical Pharmacology, the clinical application of dapagliflozin in T2DM treatment was evaluated, suggesting its safety and efficacy when used in combination with insulin, particularly for patients in whom metformin treatment fails [13]. A recent study randomly assigned 198 patients, with 195 included in the efficacy analysis (dapagliflozin group: 96 patients; placebo group: 99 patients). Eligible participants were individuals aged ≥19 years with T2DM mellitus receiving combination therapy of metformin (≥1000 mg/day, any formulation) and efpeglitinib 5 mg/day for at least 8 weeks. The trial was conducted at 26 centers in South Korea between 26 May 2020 and 23 February 2022. At week 24, dapagliflozin significantly reduced glycated hemoglobin (HbA1c) levels and improved multiple glycemic and metabolic parameters, with no significant differences in the spectrum of adverse events between the two groups [14]. Similarly, the efficacy and safety of dapagliflozin in Japanese patients with T2DM were confirmed, with significant improvements in HbA1c, fasting blood glucose, and weight loss indices compared with those in the placebo group [15]. These findings highlight the potential of SGLT2 inhibitors, such as dapagliflozin, to enhance insulin pump intensive therapy in T2DM patients.

In summary, due to the current lack of comprehensive clinical studies in the literature examining the impact of adding oral hypoglycemic agents to intensive insulin therapy on key treatment outcomes, this prospective, open‐label, controlled, parallel‐group study compared the therapeutic outcomes of intensive insulin pump therapy alone versus intensive insulin pump therapy combined with either metformin or dapagliflozin. It assessed differences in time spent in the target range, total insulin dose at the conclusion of intensive therapy, and pancreatic function. The study aimed to provide a robust evidence base for the clinical management of T2DM.

## 2. Methods

A total of 110 patients with newly diagnosed T2DM were enrolled in this single‐center randomized controlled trial. The inclusion criteria were a glycated hemoglobin level >9%, a fasting blood glucose level >11.1 mmol/L, or obvious symptoms of hyperglycemia. The exclusion criteria were severe infection, acute complications (e.g., hyperglyaemic hyperosmolar state, diabetic ketoacidosis), trauma, severe malnutrition, severe liver or kidney dysfunction (transaminase levels more than three times the normal value, eGFR <60 mL/min/1.73 m^2^), type 1 diabetes, pregnancy or lactation, and desire for pregnancy. Patients were randomized into three groups: insulin pump therapy alone, insulin pump therapy + dapagliflozin, and insulin pump therapy + metformin.

Detailed study procedures, follow‐up schedule, and recruitment/randomization methodology are provided in the Supplementary Methods.

### 2.1. Statistical Analysis

The normality of the descriptive data was tested via the Shapiro‒Wilk test. The arithmetic means and standard deviation ($\overset{―}{X}\pm s$) were used to characterize the data if the normality assumption was met; otherwise, the median and interquartile range (*M*(*P*25, *P*75)) were used. Two‐sample means were compared via *t* tests or nonparametric tests if the assumption of parametric tests for numerical data was violated. To infer whether two or more population means were equal, ANOVA or the Kruskal‒Wallis H test was applied according to the distribution of the data. Multiple comparisons were made after the significance level was adjusted by the Bonferroni method if the population means were not identical. Differences in categorical data were examined via the chi‐square test or nonparametric test based on the nature of the data. ANOVA for repeated measures was performed to infer whether the main outcome indicator had improved compared with the baseline. Greenhouse–Geisser estimation was used if the sphericity assumption was not met. Because of the skewed distribution of insulin and C‐peptide data, logarithmic transformation was conducted before ANOVA. All the data were independently input via EpiData by two researchers, and computations were performed using SPSS 19.0. A complete‐case analysis was selected to handle missing values. All tests were 2‐tailed; *p* < 0.05 was considered significant.

Treatment adherence was monitored throughout the study. For the insulin pump, adherence was inherent to the inpatient setting and was monitored by nursing staff. Adherence to oral medications (dapagliflozin and metformin) was assessed via direct questioning and pill count by the study investigators during the daily inpatient visits.

## 3. Results

### 3.1. Participant Flow and Adherence

**Figure 1:**
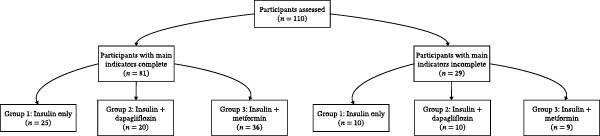
CONSORT flow diagram of participant enrollment and follow‐up.

The flow of participants through the study is presented in a CONSORT diagram (Figure 1). Briefly, a total of 110 participants were enrolled and randomized. Of these, 81 participants constituted the per‐protocol population with complete data for the main indicators (time to achieve glycemic targets, total insulin dose at the end of intensive therapy, and pancreatic function as assessed by fasting and postprandial C‐peptide and insulin levels), distributed as Group 1 (insulin only, *n* = 25), Group 2 (insulin plus dapagliflozin, *n* = 20), and Group 3 (insulin plus metformin, *n* = 36). The remaining 29 participants, who provided incomplete primary endpoint data, were distributed across the groups (Group 1: *n* = 10; Group 2: *n* = 10; Group 3: *n* = 9). Statistical comparison of baseline characteristics between participants with complete and incomplete data revealed no significant differences in observed variables (See Table 1), though this does not definitively establish the missing data mechanism (MCAR).

**Table 1:** Baseline characteristics of participants with or without main indicators complete in three groups ^∗^.

Variable	Participants with main indicators complete			*p*‐Value^a^	Participants with main indicators incomplete			*p*‐Value^a^	*p*‐Value^b^
	Group 1 (*n* = 25)	Group 2 (*n* = 20)	Group 3 (*n* = 36)		Group 1 (*n* = 10)	Group 2 (*n* = 10)	Group 3 (*n* = 9)		
Age (years)				0.989				0.268	0.571
<40	6 (30.00%)	4 (20.00%)	10 (50.00%)		3 (60.00%)	1 (20.00%)	1 (20.00%)		
40–59	9 (32.14%)	7 (25.00%)	12 (42.86%)		1 (11.11%)	3 (33.33%)	5 (55.56%)		
≥60	10 (30.30%)	9 (27.27%)	14 (42.42%)		6 (40.00%)	6 (40.00%)	3 (20.00%)		
Sex				0.733				0.144	0.377
Male	16 (31.37%)	11 (21.57%)	24 (47.06%)		5 (23.81%)	9 (42.86%)	7 (33.33%)		
Female	9 (30.00%)	9 (30.00%)	12 (40.00%)		5 (62.50%)	1 (12.50%)	2 (25.00%)		
BMI				0.439				0.081	0.471
<24.0	9 (25.00%)	7 (19.44%)	20 (55.56%)		1 (11.11%)	4 (44.44%)	4 (44.44%)		
24.0–27.9	9 (32.14%)	9 (32.14%)	10 (35.71%)		5 (41.67%)	2 (16.67%)	5 (41.67%)		
≥28.0	7 (41.18%)	4 (23.53%)	6 (35.29%)		4 (50.00%)	4 (50.00%)	0 (0.00%)		
WHR^c^				0.429				1.000	1.000
Central obesity	3 (30.00%)	1 (10.00%)	6 (60.00%)		1 (33.33%)	1 (33.33%)	1 (33.33%)		
Noncentral obesity	21 (30.88%)	19 (27.94%)	28 (41.18%)		9 (36.00%)	9 (36.00%)	7 (28.00%)		
Hypertension^d^				0.703				0.266	0.191
Yes	17 (34.69%)	11 (22.45%)	21 (42.86%)		5 (38.46%)	6 (46.15%)	2 (15.38%)		
No	8 (25.00%)	9 (28.13%)	15 (46.88%)		5 (31.25%)	4 (25.00%)	7 (43.75%)		

*Note:* Glycosylated hemoglobin has little data in the incomplete dataset, and so does fasting blood glucose. Thus, baseline comparisons are conducted only in the complete dataset, which is the dataset with main indicators complete.

^∗^Group 1, Group 2, and Group 3 denote three different treatment groups in this trial, namely insulin only, insulin plus dapagliflozin, and insulin plus metformin, respectively.

^a^Chi‐squared or Fisher–Freeman–Halton exact probability tests are applied to infer whether proportions differ significantly in the complete or incomplete dataset, respectively, considering age, sex, BMI, etc.

^b^Chi‐squared test is adopted to verify that the presence of missing data on main indicators is unrelated to the baseline characteristics.

^c^WHR refers to waist‐hip ratio, and is categorized into central obesity if WHR > 0.9 or WHR > 0.85 for male or female, respectively. Three objects did not report their WHR in the main indicator variable complete dataset.

^d^Hypertension is defined as SBP ≥140 mmHg or DBP ≥90 mmHg.

The study is a single‐center randomized clinical trial conducted from July 2022 to June 2023 in the Endocrinology Clinic, The Third Affiliated Hospital of Guangzhou Medical University, Guangzhou, China. A total of 110 diabetes patients were recruited. However, observations with missing values for any of the main indicator variables listed in Table 2 were grounds for patient exclusion. As a result, 81 (73.67%) valid patients remained. Table 1 presents the characteristics of the complete and incomplete datasets, demonstrating balanced distribution across experimental groups for all three subject cohorts. All *p*‐values (assessing associations between missing data and baseline characteristics) exceeded 0.05 (age *p* = 0.571, sex *p* = 0.377, BMI *p* = 0.471, WHR *p* = 1.000, hypertension *p* = 0.191), indicating that the occurrence of “primary indicator data missing” in subjects was unrelated to baseline characteristics such as age, gender, BMI, central obesity status, or history of hypertension. The nonsignificant test results in the last column of Table 1 suggest that the missing data may be missing completely at random (MCAR), though other mechanisms cannot be ruled out.

**Table 2:** Main outcome indicators at baseline and endpoint in the three groups ^∗^.

Variables	Time^a^	Group 1 (*n* = 25)	Group 2 (*n* = 20)	Group 3 (*n* = 36)	*p*‐Value
Glycosylated hemoglobin	B	11.52% ± 2.12%^b^	11.13% ± 1.52%	11.71% ± 1.82%	0.534
	E	9.98% ± 2.11%	10.00% ± 1.23%	10.59% ± 1.89%	0.333
Blood glucose					
Fasting	B	9.96 ± 2.62	8.29 ± 2.13	9.68 ± 2.90	0.088
	E	6.91 ± 1.29	6.27 ± 1.05	7.26 ± 1.35	0.023
0.5 h postprandial	B	14.51 ± 3.42	13.73 ± 3.81	14.71 ± 3.46	0.605
	E	9.97 ± 2.26	10.06 ± 1.79	10.85 ± 2.66	0.289
2 h postprandial	B	21.93 ± 3.66	19.69 ± 4.51	22.03 ± 3.03	0.054
	E	14.44 ± 4.24	14.19 ± 3.02	15.83 ± 3.79	0.208
Insulin					
Fasting	B	9.60 (7.35, 11.20)^c^	10.20 (8.48, 13.93)	8.50 (6.70, 10.40)	0.068
	E	7.50 (5.90, 8.75)	8.05 (5.34, 11.98)	8.60 (6.60, 10.35)	0.490
0.5 h postprandial	B	8.50 (6.90, 13.10)	10.70 (8.78, 18.50)	9.00 (6.30, 13.90)	0.138
	E	13.90 (8.75, 16.00)	15.29 (12.40, 23.83)	13.62 (8.20, 23.63)	0.293
2 h postprandial	B	12.60 (9.40, 18.10)	19.65 (14.50, 29.50)	10.35 (8.63, 22.43)	0.007
	E	29.30 (18.45, 45.10)	31.10 (19.95, 44.45)	27.65 (18.40, 34.30)	0.519
C‐peptide					
Fasting	B	374.00 (212.00, 513.00)	475.50 (248.25, 679.75)	313.00 (225.75, 515.25)	0.248
	E	445.00 (386.00, 661.50)	413.50 (364.75, 573.00)	503.00 (296.00, 739.00)	0.912
0.5 h postprandial	B	500.00 (301.50, 636.00)	577.50 (481.50, 765.50)	449.50 (263.75, 795.00)	0.277
	E	754.00 (557.50, 960.50)	778.50 (619.00, 1128.50)	825.00 (478.25, 1324.75)	0.811
2 h postprandial	B	974.00 (644.00, 1513.00)	1254.00 (1001.25, 1779.50)	900.50 (524.00, 1627.25)	0.053
	E	1654.00 (1363.50, 2702.50)	2128.50 (1276.00, 2760.50)	1887.00 (1290.00, 2419.00)	0.766

^∗^Group 1, Group 2, and Group 3 denote three different treatment groups in this trial, namely insulin only, insulin plus dapagliflozin, and insulin plus metformin, respectively.

^a^The time column refers to the measured time when the experiment is done, B indicates the baseline, while E indicates the endpoint.

^b^Median, 25th, and 75th percentiles are expressed in the form of (*M*(*P*25, *P*75)) for describing the non‐normal data. The Krustal–Wallis H test is employed to test the non‐normal data.

^c^Arithmetic mean and standard deviation are expressed in the form of ($\overset{―}{X}\pm s$) for describing the normal data. ANOVA is employed to test the normal data.

Table 2 shows the primary outcome indicators at baseline and at the endpoint. In addition to the 2‐h postprandial insulin levels, there were no significant differences in the remaining nine indicators (*p* > 0.05) at baseline. For 2‐h postprandial insulin levels, pairwise comparisons adjusted by the Bonferroni method revealed that insulin plus dapagliflozin (Group 2) was more effective than insulin (Group 1) or insulin plus metformin (Group 3) at the baseline time point.

As shown in Table 2, the 0.5‐h (decreased to 9.97–10.85) or 2‐h (decreased to 14.19–15.83) postprandial levels of glycosylated hemoglobin and blood glucose decreased, whereas the 0.5‐h (rose to 754.00–825.00) or 2‐h (rose to 974.00–1254.00) postprandial levels of C‐peptide and insulin increased. All three treatment groups effectively improved glycemic control (reducing both glycated hemoglobin and fasting/postprandial blood glucose levels) and pancreatic β‐cell function (elevating C‐peptide) in patients with T2DM. Among these, insulin combined with dapagliflozin demonstrated superior efficacy in controlling fasting blood glucose (*p* = 0.023 between endpoint groups). For the remaining parameters (HbA1c, postprandial glucose, insulin, C‐peptide), the three groups showed comparable effects with no significant intergroup differences.

The ANOVA for repeated measures, with logarithmic transformation to correct for skewness in the insulin and C‐peptide data, indicated significant changes in the glycated hemoglobin, blood glucose, and C‐peptide levels over time (Table 3). Specifically, for glycated hemoglobin, the main effect of time was statistically significant (*F* = 73.851, *p* < 0.001), whereas the main effect of treatment (*F* = 0.862, *p* = 0.426) and the treatment–time interaction effect (*F* = 0.925, *p* = 0.401) were both statistically insignificant. This indicates that HbA1c levels decreased significantly throughout the treatment period regardless of the therapeutic regimen employed, yet no differences were observed between the three treatment groups in their efficacy or rate of HbA1c reduction. Regarding blood glucose levels, all three time points (fasting, 0.5‐h postprandial, and 2‐h postprandial) exhibited highly significant main effects of time (*p* < 0.001), with *F*‐values of 61.932, 62.369, and 154.359, respectively. This outcome underscores that intensive insulin pump therapy effectively reduces blood glucose levels across all postprandial phases, with the most pronounced improvement observed in the 2‐h postprandial phase. Regarding treatment main effects, only fasting blood glucose showed statistical significance (*F* = 4.384, *p* = 0.016), whilst no significant differences were observed for 0.5‐h postprandial glucose (*F* = 1.331, *p* = 0.270) or 2‐h postprandial glucose (*F* = 2.941, *p* = 0.059). Post hoc comparisons further confirmed that the insulin plus dapagliflozin group exhibited a greater reduction in fasting blood glucose compared to the other two groups. No significant treatment‐by‐time interaction effects were detected for any glucose parameters (*p* > 0.05), indicating no difference in the rate of blood glucose reduction among the three groups.

**Table 3:** ANOVA for repeated measures.

Variables	Treatment^a^		Time^b^		Treatment ^∗^ Time^c^	
	F statistic	*p* value	F statistic	*p* value	F statistic	*p* value
Glycosylated hemoglobin	0.862	0.426	73.851	<0.001	0.925	0.401
Blood glucose						
Fasting	4.384	0.016	61.932	<0.001	0.797	0.454
0.5 h postprandial	1.331	0.270	62.369	<0.001	0.252	0.778
2 h postprandial	2.941	0.059	154.359	<0.001	1.175	0.314
Insulin^d^						
Fasting	0.968	0.384	14.012	<0.001	5.148	0.008
0.5 h postprandial	2.017	0.140	25.366	<0.001	0.263	0.769
2 h postprandial	3.033	0.054	51.439	<0.001	2.542	0.085
C‐peptide						
Fasting	0.936	0.397	8.663	0.004	2.577	0.082
0.5 h postprandial	1.307	0.277	36.228	<0.001	0.964	0.386
2 h postprandial	1.776	0.176	59.596	<0.001	2.008	0.141

^a^Treatment refers to the main effect of the treatment factor, which has three levels, namely insulin only, insulin plus dapagliflozin, and insulin plus metformin.

^b^Time refers to the main effect of the time factor, which has two levels, namely the measurement on the 1st day and that on the 14th day.

^c^Treatment  ^∗^ Time here refers to the interaction of the treatment and time.

^d^Insulin and C‐peptide have been logarithmic transformed to improve the normality.

## 4. Discussion

Our clinical study provides evidence regarding the impact of adding OADs to intensive insulin pump therapy in patients with T2DM. The key findings suggest that while all three regimens effectively improved glycemic control and pancreatic β‐cell function, the addition of dapagliflozin resulted in a greater reduction in fasting blood glucose and a faster decline in fasting insulin levels compared to metformin or insulin alone.

Our findings confirm that the distribution of participants across the three experimental groups was balanced, thereby supporting the assumption of MCAR. This strengthens the validity of our statistical analysis. Upon closer examination of the baseline characteristics, we observed no significant differences among the groups in most indicators. However, 2‐h postprandial insulin levels were higher in both the insulin + dapagliflozin and insulin + metformin groups than in the insulin‐alone group. This observation suggests the additional oral antidiabetic agents may have already affected insulin levels prior to the start of intensive therapy. We further observed that although intensive insulin pump therapy effectively improved glycemic control and pancreatic β‐cell function, the addition of oral hypoglycemic agents did not significantly alter the overall trend of these parameters. Notably, the insulin‐plus‐dapagliflozin group demonstrated superior efficacy in reducing fasting blood glucose levels and accelerating the rate of decline in fasting insulin concentrations. These findings indicate that the combination of insulin and dapagliflozin may lead to a faster decrease in fasting insulin levels than the combination of insulin and metformin.

These results are consistent with the known mechanisms of action of both dapagliflozin and metformin. Dapagliflozin, which is an SGLT2 inhibitor, facilitates glucose excretion and may improve insulin sensitivity, whereas metformin works primarily by decreasing hepatic glucose production and increasing insulin sensitivity [15, 16]. The accelerated decline in fasting insulin levels observed in the dapagliflozin group may reflect its synergistic effect with insulin pump therapy, potentially achieved by reducing insulin resistance and decreasing the demand for endogenous insulin secretion. This aligns with previous findings that SGLT2 inhibitors can reduce insulin requirements while maintaining glycemic control in patients with T2DM [17, 18]. It is important to acknowledge the limitations of our study: the open‐label design and use of post‐hoc comparisons may have introduced bias into the findings; additionally, the relatively small sample size and single‐center design may limit the generalizability of results to broader populations or other clinical settings. In a cohort of patients with T2DM undergoing intensive insulin pump therapy, the addition of dapagliflozin to the insulin pump treatment regimen may be more efficacious than the addition of metformin in improving glycemic control.

The safety profile observed in our study was consistent with the known effects of these medications. The gastrointestinal discomfort associated with metformin initiation and the potential risk of hypoglycemia with intensive insulin therapy were observed as expected [19]. Reassuringly, no new safety signals emerged from the short‐term combination of dapagliflozin with insulin pump therapy, which aligns with existing evidence on its safety profile in combination with insulin.

Furthermore, this study was primarily designed to assess efficacy endpoints over a short duration. Therefore, the safety and tolerability data, while prospectively collected, were not as comprehensively quantified as in a dedicated safety trial. The small sample size and short follow‐up period limit the ability to detect rare adverse events.

## 5. Conclusion

The addition of dapagliflozin to insulin pump therapy may be a promising strategy for achieving glycemic control in patients with T2DM. Further studies with larger sample sizes and longer follow‐up periods are needed to confirm these findings and to investigate the long‐term effects of adding dapagliflozin to insulin pump therapy.

## 6. Supplementary Methods

### 6.1. Study Procedures and Follow‐up

The total duration of the clinical study for each participant was 15 days, which included a 1‐day screening period and a 14‐day intensive treatment period. The schedule of assessments is summarized in Table 1 (or a dedicated follow‐up schedule table).

Screening visit (Day −1 to Day 0): After providing written informed consent, potential participants underwent screening assessments to verify eligibility. This included a review of medical history, physical examination, measurement of vital signs, height, weight, and laboratory tests (including HbA1c, fasting blood glucose, liver, and renal function).

Baseline visit (Day 1): Eligible participants who passed the screening were randomized into the three treatment groups. Baseline efficacy assessments, including fasting and postprandial (0.5‐h and 2‐h) blood glucose, C‐peptide, and insulin levels following a standard steamed bun meal test, were performed.

Treatment period (Day 1 to Day 14): Participants received their assigned treatments. Glycemic control was actively managed throughout this period, with insulin doses titrated according to the predefined protocol. Seven‐point self‐monitoring of blood glucose profiles was performed daily to guide therapy and ensure safety.

Endpoint visit (Day 14): All baseline efficacy assessments (fasting and postprandial blood glucose, C‐peptide, and insulin levels) were repeated.

Post‐treatment follow‐up: A follow‐up contact (e.g., telephone call) was conducted approximately 1 week after the end of the treatment period (around Day 21) to record any adverse events and provide lifestyle guidance.

### 6.2. Participant Recruitment and Randomization

Participants were recruited from the Endocrinology outpatient clinic of The Third Affiliated Hospital of Guangzhou Medical University. Consecutive eligible patients who met the inclusion and exclusion criteria and provided informed consent were invited to participate.

A randomized, controlled, parallel‐group design was employed. Eligible participants were allocated to one of the three treatment groups using a computer‐generated randomization sequence created with Microsoft Office Professional Plus 2013. The randomization list, linking sequential participant numbers (001‐090) to treatment assignments, was prepared and sealed in opaque envelopes. Upon confirming a participant’s eligibility and obtaining consent, the investigator assigned the next sequential number and opened the corresponding sealed envelope to reveal the group assignment, ensuring allocation concealment.

### 6.3. Safety and Tolerability

Adverse events were monitored throughout the 14‐day treatment period. The most observed adverse event across all groups was hypoglycemia, which was managed by adjusting the insulin pump dosage. The incidence of symptomatic hypoglycemia was comparable between the three groups (Group 1: 1.5%; Group 2: 1.4%; Group 3: 1.4%; *p* > 0.05). No severe hypoglycemic events requiring third‐party assistance were reported. One patient in the metformin group experienced transient gastrointestinal discomfort, which resolved without discontinuing the study. No genital or urinary tract infections, events of special interest for SGLT2 inhibitors, were reported in the dapagliflozin group during the short study period. No other significant adverse events or serious adverse events were observed.

## Author Contributions

Yuyi Chen and Yingjun Xie carried out study design. Naifeng Liang, Shiyun Wen, and Yaojie Zhai prepared tables. Miaoguan Peng and Yijuan Xie wrote the paper.

## Funding

This study was supported by the Medical Science and Technology Research Fund of Guangdong Province (Grant B2025401) and Guangzhou Research‐Oriented Hospital.

## Disclosure

All authors read and approved the final manuscript.

## Ethics Statement

Ethical approval was obtained for this study from the Third Affiliated Hospital of Guangzhou Medical University (2024015). Informed consent was obtained from all subjects and/or their legal guardian(s).

## Conflicts of Interest

The authors declare no conflicts of interest.

## Data Availability

Data that support the findings of this study are available from the corresponding author upon reasonable request.
